# A comparison of revision risk between lateral knee replacement and total knee replacement: results from the Dutch Arthroplasty Register

**DOI:** 10.2340/17453674.2026.46374

**Published:** 2026-09-24

**Authors:** Iris KOENRAADT-VAN OOST, Leonieke C VAN BOEKEL, Alexander HOORNTJE, Gino M M J KERKHOFFS, Paul G H MULDER, Liza N VAN STEENBERGEN, Rutger C I VAN GEENEN

**Affiliations:** 1 Foundation for Orthopedic Research, Care & Education, Amphia Hospital, Breda; 2 Department of Orthopaedic Surgery and Sports Medicine, Amsterdam Movement Sciences, Amsterdam University Medical Center, Location Academic Medical Center, University of Amsterdam, Amsterdam; 3 Amphia Academy, Amphia Hospital, Breda; 4 Dutch Arthroplasty Register (LROI), ‘s-Hertogenbosch; 5 Department of Orthopedic Surgery, Amphia Hospital, Breda, the Netherlands

## Abstract

**Background and purpose:**

Lateral knee replacement is not commonly performed. Possible explanations are the lower incidence of lateral knee osteoarthritis, relative technical complexity, and different biomechanics, which may affect failure modes and survival rates. We aimed to analyze the difference in risk of revision between lateral and total knee replacements (TKRs) using the Dutch Arthroplasty Register data.

**Methods:**

From 2007 to 2021, procedures were selected from the Dutch Arthroplasty Register. Unique patients based on their first procedure were identified. Procedures performed between 2014 and 2021 were analyzed separately because BMI and Charnley score were collected from 2014 onwards. We used ATT (Average Treatment effect on the Treated) weights based on the propensity score to estimate the average treatment effect in the lateral knee replacement group. The endpoint was revision, censored for death. Kaplan–Meier survival analyses were performed in propensity-based ATT-weighted cohorts of lateral knee replacement and TKR patients. Weighted univariable Cox regression models were used to estimate revision hazard ratios (HR).

**Results:**

In the total cohort, 847 lateral knee replacements and 240,047 TKR patients were included. In the subgroup, 560 lateral knee replacements and 155,621 TKR patients were included. The risk of revision in both the total cohort and the subgroup was higher for lateral knee replacements compared with TKRs with an ATT-adjusted HR for the total cohort of 2.15 (95% confidence interval [CI] 1.74–2.64, P < 0.001) and for the subgroup an ATT-adjusted HR of 1.55 (CI 1.08–2.21, P = 0.02). Comparison of these ATT-weighted effects with the conditional effect estimates from a regular multivariable Cox proportional hazards regression did not show large differences in this study.

**Conclusion:**

This study showed a significantly higher risk of revision for lateral knee replacements compared with TKRs.

Medial or lateral knee replacement (KR) is an attractive option as the native biomechanics of the knee are largely preserved, whereas in total knee replacement (TKR) the anterior cruciate ligament is sacrificed [1]. This might contribute to better postoperative clinical outcome following medial or lateral KR compared with TKR [2]. Furthermore, a lower risk of complications and lower mortality have been reported [3,4]. Although the use of medial and lateral KR is increasing, several studies reported that both options are still underutilized [5,6]. One reason might be the ongoing debate regarding lateral KR long-term survival rates, which vary widely across studies.

While the medial KR is increasingly being adopted, the implementation of lateral KR is lagging behind but has been shown to be feasible in a fast-track setting [7]. The known difficulties with valgus deformities and the frequently reported bearing dislocations could play an important role in this underutilization of the lateral KR [8,9].

However, improvements in implant designs and surgical techniques, as well as a better understanding of patient indications over the last decade, have led to improved survival of lateral KR [10,11]. In a recently published study with Dutch Arthroplasty Register data, Burger et al. reported a similar survivorship of lateral and medial KR [12]. As TKR is the current alternative to lateral in valgus knee osteoarthritis, a comparison of survival rates of lateral KR and TKR is needed. The aim of our registry data study is to directly compare the risk of revision and reasons for revision for lateral KR patients with TKR patients.

## Methods

### Study design

Data was obtained from the Dutch Arthoplasty Register (LROI). The LROI database contains data on orthopedic joint implants in the Netherlands since 2007. The completeness of the LROI database in 2025 was more than 98% for all knee arthroplasty (primary as well as revision surgery), with coverage of all hospitals in the Netherlands [13,14].

The study is reported according to STROBE guidelines.

### Study population

We selected all primary lateral KR and TKR procedures for the diagnosis osteoarthritis in the period 2007–2021. A subgroup was created with procedures from 2014 onwards, when body mass index (BMI) and Charnley score were additionally collected in the LROI. For patients with 2 procedures, the first procedure was chosen. The incomplete patients were then removed from the total cohort and from the subgroup.

### Data

Data was gathered regarding surgical characteristics (e.g., type of procedure [TKR or lateral KR], year of operation, fixation type, bearing type, hospital volume [TKR + medial and lateral KR]) and patient characteristics (e.g., age at the time of surgery, sex, and American Society of Anesthesiologists Physical Status [ASA] score).

### Survival, reason for revision, and follow-up

Follow-up was defined as time between primary procedure and revision, death, or end date of follow-up (January 1, 2022). The endpoint of interest was revision, implying that death (while revision-free) was considered a censoring cause along with the above end date of follow-up. Revision was defined as every change (placement, replacement, removal, or addition) of 1 or more components of the knee prosthesis. Total revision-free follow-up and revisions across the patients amounted to 1,438,423 and 4,380 person-years with respectively 10,746 and 95 revisions in the respective TKR and lateral KR group. The median follow-up of the TKR and lateral KR group was 5.6 (interquartile range [IQR] 2.9–8.9) and 4.0 (IQR 1.6–8.0) years for the total cohort and 3.9 (IQR 2.1–5.8) and 2.6 (IQR 1.3–4.9) years for the subgroup cohort.

### Statistics

2 separate analyses were performed on the total cohort (2007–2021) and on a subgroup (2014–2021). We used ATT (Average Treatment effect on the Treated) weights based on the propensity score.

**Figure 1:**
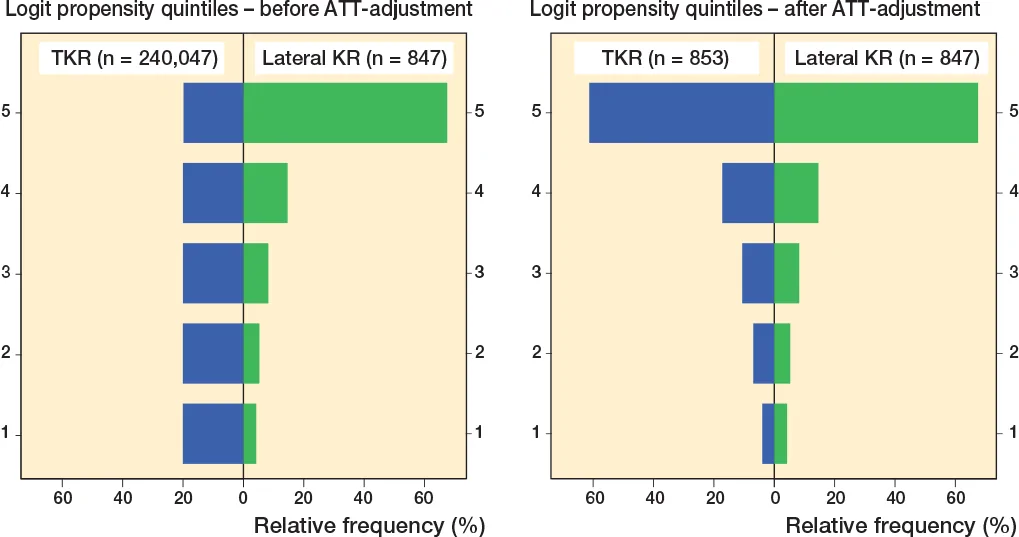
Matching of the logit of the propensity score between lateral KRs and TKRs in the total cohort (A) before and (B) after ATT adjustment (n = 853 in TKR group is sum of the weights).

Within the population of patients eligible for a knee operation, the type of operation chosen for a patient is based on indication by the treating orthopedic surgeon rather than on a free choice for all patients. This supports the use of propensity-based ATT weights with lateral KR as the treated group rather than the regular use of ATT weights in the entire population. The average treatment effect of lateral KR relatively to TKR can then be estimated counterfactually within this lateral KR group by comparing the actual lateral KR treatment with the TKR treatment that this group would have counterfactually undergone. Moreover, given the relatively small lateral KR group, using ATT weights instead may lead to biased treatment effects [15]. According to ATT weighting, patients in this small lateral KR group were not weighted. Patients in the very large TKR group were weighted by their propensity odds. After this ATT weighting the distribution of the pre-selected independent variables for revision (as entered in the propensity score) appeared to match very well with the observed distribution of these variables in the small lateral KR group. Also, the sum of the weights was approximately equal to the number of patients in the lateral KR group (853 vs 847). This was also the case for the subgroup: 563 vs 560. For quantifying the matching, we calculated the absolute value of the standardized mean difference (SMD) between the 2 treatments before and after the ATT weighting. The SMD decreased substantially to a value near zero after weighting (Table 1). This calculation was done for each variable separately, as well as for the logit of the propensity score. The logit is the linear predictor in the propensity score (a linear combination of all variables). As a rule of thumb an absolute SMD < 0.1 is considered to denote a good match, which appeared to hold for all pre-selected variables after the matching (Table 1). Variables with an already good match beforehand were not deleted from the analysis as they still can be good predictors for the revision rate theoretically. For a graphical illustration of the result of ATT weighting in the total cohort we categorized the continuous logit of the propensity score into 5 quintiles (Figure 1). Before weighting the distribution of the logit was nearly uniform in the very large TKR group (Figure 1A). After applying the propensity odds as weights in the TKR group (Figure 1B), the distribution of the logit in the TKR group matched the distribution in the lateral KR group. The subgroup had a similar good matching result (not shown).

**Table 1:** Absolute standardized mean differences (SMD) between the 2 treatments before and after ATT weighting in the total cohort and in the subgroup

Variable	Total cohort		Subgroup	
	SMD before	SMD after	SMD before	SMD after
Age	0.671	0.003	0.552	0.014
Hospital volume	0.312	0.004	0.089	0.020
Sex	0.135	0.000	0.216	0.005
Cementless fixation	0.060	0.001	0.003	0.002
Hybrid fixation	0.710	0.011	1.040	0.011
ASA II score	0.247	0.008	0.054	0.011
ASA III/IV score	0.262	0.002	0.271	0.004
BMI			0.436	0.010
Charnley score			0.460	0.004
Logit	1.201	0.013	1.431	0.019

ASA = American Society of Anesthesiologists Physical Status classification; BMI = body mass index.

The propensity score was calculated as the probability outcome of a logistic regression model with lateral KR treatment as dependent variable. The choice of the independent variables included was based on their relevance as determinants of the revision rate: age, sex, ASA, fixation type (cemented, cementless, or hybrid), hospital volume (all primary knee operations in the patient’s operation year considered), BMI and Charnley score (as linear trend). In order to define the proxy variable “hospital volume” for the routine that the hospital had at the time of operation we calculated the total number of primary knee operations per hospital during the year that the patient was operated. This number entered the logistic regression model after logarithmic transformation

According to ATT weighting, the lateral KR patients were not weighted (weight = 1). The TKR patients were weighted by their propensity odds. This turns the TKR group into a proper control group in which the distribution of all selected variables matches with that in the lateral KR group, as is the case with randomization. The advantage of this approach is that the revision hazard ratio of lateral KR to TKR can be estimated univariably by a weighted Cox proportional hazards survival analysis with treatment as the only independent variable. Due to this “pseudo randomization” the estimated treatment effects are more properly interpretable as causal effects.

To visualize the effect of ATT weighting graphically, we categorized the distribution of the logit of the propensity score into quintiles and compared this distribution between TKR and lateral KR before and after ATT weighting. For a quantitative judgment of differences between the TKR and the lateral KR group in the logit (as continuous variable) as well as in all variables separately of the propensity score (of which the logit is a linear combination), we calculated the standardized mean difference (SMD). This was done before and after ATT weighting. The SMD is defined as the difference in means between 2 groups, expressed in standard deviation (SD) units, where the SD equals the square root of the average of both variances. Weighting affects only the mean difference. The SD is calculated without applying the weights.

Kaplan–Meier survival analyses were performed to determine revision-free probability at the 4-year and 6-year follow-up for the total cohort and the subgroup. For the total cohort, the revision-free probability at 8-year follow-up could also be determined, because the follow-up was longer (2007–2021). These survival analyses were performed with revision as endpoint.

Univariable Cox regression analyses with weighted cohorts were used to estimate hazard ratios (HRs) with a 95% confidence interval (CI) to compare the risk of revision between lateral KR and TKR.

P values < 0.05 were considered significant. The statistical package SPSS (version 25; IBM Corp, Armonk, NY, USA) and R studio (version 2021.9.1.372; R Foundation for Statistical Computing, Vienna, Austria) were used for all statistical analyses.

### Ethics, registration, data sharing plan, funding, and disclosures

As the study was based on registry data, ethical approval was not needed. This study received no funding and the authors declare no conflicts of interest regarding this study. Complete disclosure of interest forms according to ICMJE are available on the article page, doi: 10.2340/17453674.2026.46374

## Results

**Figure 2:**
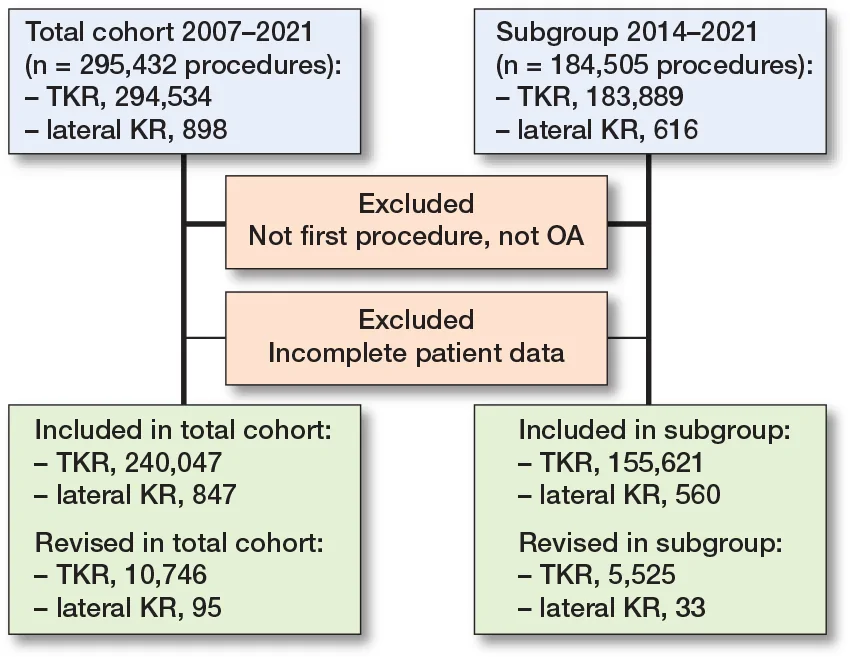
Study flow diagram. Patients were included with diagnosis osteoarthritis (OA) and for patients with 2 procedures, the first procedure was chosen. 3.3% incomplete patients in the total cohort and 3.1% incomplete patients in the subgroup were excluded.

98 hospitals performed 295,432 procedures for the diagnosis osteoarthritis from 2007 to 2021. By selecting the earlier procedure of patients with 2 procedures in the data set and removing the incomplete patients from the total cohort (3.3%) and from the subgroup (3.1%), we identified 240,894 unique patients, of whom 847 received a lateral KR procedure. The number of revisions in the total cohort was 10,841 during a total of 1,442,803 person-years, of which 95 in the lateral KR group during 4,380 person-years. The subgroup of patients operated on from 2014 to 2022 consisted of 156,181 patients, of whom 560 had a lateral KR. The number of revisions in this subgroup was 5,525 during 609,655 person-years, of which 33 in the lateral KR group during 1,752 person-years (Figure 2).

The patient characteristics relevant for the revision rate showed an overall lower age, less male sex, lower ASA, 30% hybrid fixation and less cemented fixation, more revisions, and in the subgroup also a lower BMI and lower Charnley score in the lateral knee replacement group (Table 2).

**Table 2:** Patient characteristics of the total cohort and the subgroup. Values are count (%) unless otherwise specified

Factor	Total cohort 2007–2021		Subgroup 2014–2021	
	Lateral KR n = 860	TKR n = 248,247	Lateral KR n = 590	TKR n = 160,627
Incomplete patients	13 (1.5)	8,200 (3.3)	30 (5.1)	5,006 (3.1)
Complete patients	847 (99)	240,047 (97)	560 (95)	155,621 (97)
Age, mean (SD)	62 (11)	69 (9.1)	63 (11)	69 (8.9)
Missing, n	1	253	1	82
Male sex, (%)	(30)	(36)	(27)	(37)
Missing, n	1	406	0	101
BMI, mean (SD)	–	–	27.6 (4.2)	29.6 (4.9)
Missing, n			18	2,668
ASA grade				
I	319 (37)	40,430 (17)	149 (25)	20,568 (13)
II	462 (54)	160,018 (66)	379 (65)	107,658(67)
III–IV	71 (8.3)	40,624 (17)	60 (10)	32,258 (20)
Missing, n	8	7,175	2	143
Charnley score	–	–		
A			424 (74)	71,490 (45)
B1			98 (17)	59,813 (38)
B2			33 (5.7)	21,856 (14)
C			19 (3.3)	5,038 (3.2)
Missing, n			16	2,430
Fixation type				
Cemented	574 (67)	222,665 (91)	321 (54)	148,699 (93)
Cementless	30 (3.5)	11,658 (4.7)	23 (3.9)	6,301 (3.9)
Hybrid	253 (30)	11,271 (4.5)	245 (42)	5,488 (3.4)
Missing, n	3	2,653	1	139
Hospital volume median (range)	288 (2–714)	288 (1–795)	322 (23–633)	303 (1–795)
Missing, n	0	0	0	0
Revision	96 (11)	11,182 (4.5)	40 (6.8)	5,731 (3.6)
Missing, n	2	0	0	0

For abbreviations, see Table 1 and (T)KR = (total) knee replacement.

The number of lateral KRs increased over the years from 28 in 2007 to 111 in 2021. It is a relatively rare treatment option (fewer than 5 per 1,000 knee arthroplasties) [14]. Also, the number of lateral KRs per hospital was low. In the last 3 years (2019–2021) the mean number of lateral KRs was around 5 per year per hospital with a range between 1 and 21 lateral KRs in the 73 hospitals that performed lateral KRs.

### Outcome

In the total cohort, the 4-year, 6-year, and 8-year revision-free probabilities for the TKRs were, respectively, 95.9% (CI 95.9–96.0), 95.2% (CI 95.1–95.3), and 94.5% (CI 94.4–94.6). For the lateral KRs these probabilities were 90.8% (CI 88.6–93.0), 86.8% (CI 84.1–89.7), and 84.7% (CI 81.6–87.9) (Table 3). In the subgroup, the 4-year and 6-year probabilities for the TKRs were, respectively, 96.0% (CI 95.9–96.1), and 95.3% (CI 95.2–95.5) and for the lateral KRs the probabilities were 92.1% (CI 89.3–95.0), and 90.3% (CI 86.8–93.8) (Table 3).

**Table 3:** Results from the 4-, 6-, and 8-year Kaplan–Meier survival analysis and the weighted univariable Cox regression on the total cohort 2007–2021 and the results from the 4- and 6-year Kaplan–Meier survival analysis on the subgroup 2014–2021

Factor	n	Revision rate per 1,000 person-years	4-year revision-free survival (CI) %	6-year revision-free survival (CI) %	8-year revision-free survival (CI) %	HR (CI)	P value
Total cohort							
TKR	240,047	7.5	95.9 (95.9–96.0)	95.2 (95.1–95.3)	94.5 (94.4–94.6)	reference	
Lateral KR	847	21.7	90.8 (88.6–93.0)	86.8 (84.1–89.7)	84.7 (81.6–87.9)	2.15 (1.75–2.64)	< 0.001
Subgroup							
TKR	155,621	9.0	96.0 (95.9–96.1)	95.3 (95.2–95.5)	–	reference	
Lateral KR	560	18.8	92.1 (89.3–95.0)	90.3 (86.8–93.8)	–	1.55 (1.08–2.21)	0.02

For abbreviations, see Table 2 and CI = 95% confidence interval; HR = hazard ratio.

The crude revision-free mortality ratio in TKR relatively to lateral KR is 2.87 (CI 2.35–3.52) in the total cohort and 1.98 (CI 1.41–2.79) in the subgroup.

**Figure 3:**
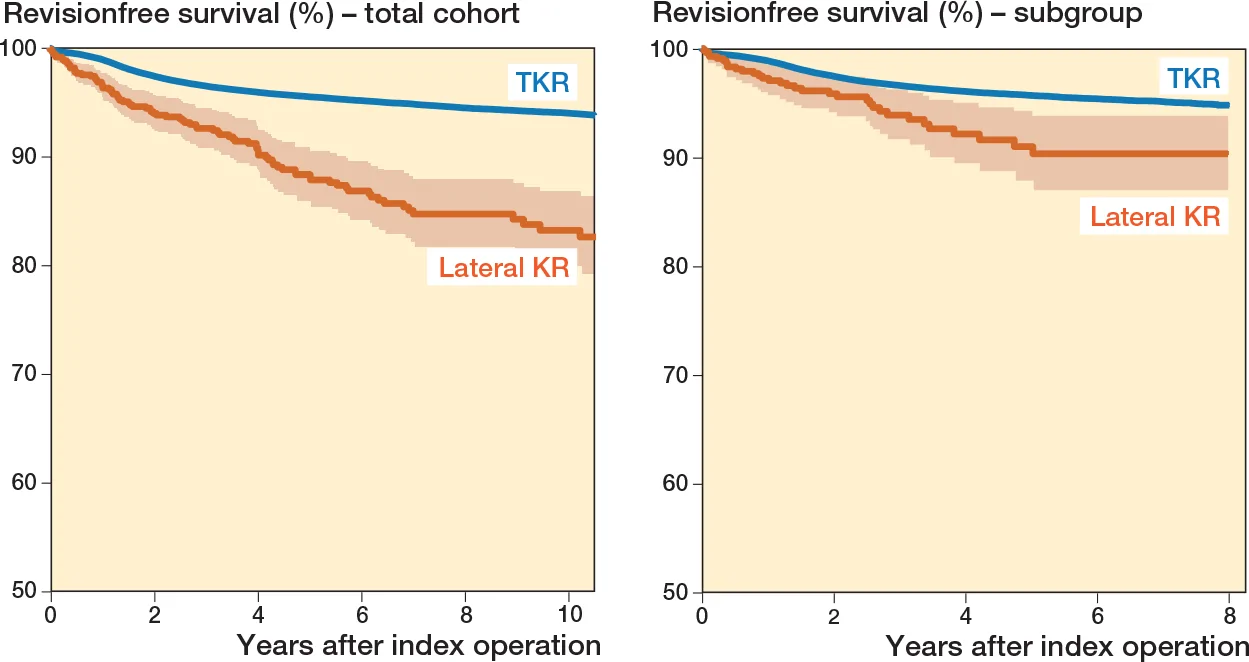
Weighted Cox proportional hazard regression survival curve for the total cohort 2007–2021 and for the subgroup 2014–2021 (additionally adjusted for BMI and Charnley score). TKR: total knee replacement. Lateral KR: lateral knee replacement.

After adjustment through ATT weighting, the estimated effects (HR) were reduced compared with the crude effects, in the total cohort as well as in the subgroup. The weighted univariable Cox regression showed a significantly higher revision rate in the lateral KRs relatively to the TKRs in the total cohort with an HR of 2.15 (CI 1.75–2.64; P < 0.001) and in the subgroup with an HR of 1.55 (CI 1.08–2.21; P = 0.02) (Figure 3).

Adjustment of the treatment effects through conditioning in a regular multivariable Cox proportional hazards regression analysis, where the same variables as used in the propensity score along with treatment enter the Cox proportional hazards model, showed results that differ only slightly from the marginal results in the case of ATT weighting. In the total cohort and in the subgroup the conditional HRs were respectively 2.05 (CI 1.67–2.52) and 1.53 (CI 1.08–2.16).

### Reasons for revision

Most frequently registered reasons for revision in lateral KR in the total cohort were progression of osteoarthritis, polyethylene wear, malalignment, instability of the prosthesis, and loosening of the femoral or tibial component. These reasons for revision were more common in lateral KRs compared with the TKRs (Table 4). In the subgroup, the same reasons for revision were reported (Table 4). Most frequently registered reasons for revision for TKR in both cohorts were patellar pain, instability and loosening, and infection.

**Table 4:** Reasons for revision in the total cohort (2007–2021) and in the subgroup (2014–2021). One revision can have multiple reasons for revisions. Values are count (%)

Reasons for revision	Total cohort		Subgroup	
	TKR n = 204,047	Lateral KR n = 847	TKR n = 155,621	Lateral KR n = 560
Arthrofibrosis	607 (5.4)	2 (2.1)	439 (7.7)	2 (5.0)
Bearing dislocation	1 (0)	0 (0)	1 (0)	0 (0)
Infection	2,229 (20)	6 (6.3)	1,424 (25)	4 (10)
Instability	3,275 (29)	20 (21)	1,686 (29)	13 (33)
Loose femur	743 (6.6)	8 (8.3)	284 (5.0)	2 (5.0)
Loose patella	147 (1.3)	0 (0)	61 (1.1)	0 (0)
Loose tibia	2,474 (22)	13 (14)	1,043 (18)	5 (13)
Malalignment	1,705 (15)	12 (3)	781 (14)	6 (15)
Other reason	1,153 (10)	15 (16)	485 (8.5)	8 (20)
Patellar dislocation	371 (3.3)	0 (0)	215 (3.8)	0 (0)
Patellar pain	3,752 (34)	9 (9.4)	1,750 (31)	5 (13)
Periprosthetic fracture	252 (2.3)	1 (1.0)	138 (2.4)	0 (0)
Polyethylene wear	380 (3.4)	6 (6.3)	97 (1.7)	2 (5.0)
Progression OA	65 (0.6)	48 (50)	29 (0.5)	9 (23)
Revision removal	301 (2.7)	2 (2.1)	98 (1.7)	0 (0)

OA = osteoarthritis.

## Discussion

In this study we compared the risk of revision between lateral KRs and TKRs and showed that lateral KRs have a 2.2 times higher risk of revision than TKRs. In the subgroup analysis, adjusting for BMI and Charnley score, the HR was reduced to 1.55.

This is a unique study, as we compared survival rates of lateral KR with TKR, which is the current best alternative for lateral KR in patients with lateral osteoarthritis. Previous studies only compared medial with lateral KRs, which is of limited value as medial and lateral KR procedures have different indications and biomechanics [12,16]. A problem with the comparison between lateral KRs and TKRs may be the lower threshold for revision for lateral KR. Tay and colleagues reported that KRs have a double lifetime risk of revision compared with TKR [17]. This is in line with our results. Several factors play a role in the higher risk of revision of lateral KR. Leg alignment can affect the osteoarthritis pattern and as a result most patients who were treated with a lateral KR had a valgus leg alignment. However, post-lateral meniscectomy patients with a neutral or slight varus alignment, instead of the valgus alignment most patients have, may be more prone to develop medial osteoarthritis. Furthermore, a lateral parapatellar approach provides limited visualization of the medial compartment and is more difficult to enlarge for conversion to TKR. Thus, the threshold for intraoperative conversion to a TKR is relatively high compared with a medial KR procedure. Also, overstuffing of the lateral compartment due to lateral ligamentous laxity is common, resulting in progression of medial osteoarthritis [18,19].

A comparison of lateral KR and TKR performed in patients with a valgus deformity is likely the best way to compare these cohorts, as performing a TKR for valgus deformity continues to be a challenge for surgeons. Only 10% of patients with osteoarthritis have a valgus deformity [20]. The most challenging factors are restoration of neutral mechanical axis and correct ligament balance, which is of importance for the longevity of the prosthesis [21]. Therefore, a future study comparing these 2 groups would be valuable as has been previously described for medial KR vs TKR for anteromedial osteoarthritis in a study by Witjes et al. They concluded that patients treated with TKR for anteromedial osteoarthritis showed better knee-specific function scores and satisfaction scores compared with the patients treated with TKR for other wear patterns [22]. Possibly, TKR for valgus osteoarthritis has survival rates that are more in line with the survival rates we observed for lateral KR.

Our relatively good survival for lateral KRs is in line with other studies showing a 10-year survival of 85% [23]. Furthermore, valgus knees undergoing TKA have a 2 times higher risk of revision compared with those in varus [24]. Nevertheless, a high dislocation rate of 4% was found and therefore they recommended an intraoperative assessment of the stability of the bearing. The same conclusion was drawn by Mohammad et al. in a registry study [25]. The high dislocation rate observed in these studies was in line with our findings, as bearing dislocation was one of the most frequent reasons for revision (20.1% of the revisions were due to bearing dislocation; before adding this item, the orthopedic surgeons used instability to register bearing dislocation).

There are a few possible explanations for the discrepancy in findings in the total cohort compared with the subgroup (better survival and lower HR in the subgroup). The first could be the increasing lateral KR volume in the period between 2014 and 2021 due to better knowledge of indications (patient selection criteria) and larger surgeon volumes due to better experience. Higher hospital and surgeon volume of KR results in better survival [26-28]. To achieve a high volume of lateral KRs, knowledge of the right indications and patient selection is important, as has also been concluded by Bunyoz et al. [29].They also stated that starting up the practice of lateral KR is safe and efficient for surgeons who already have experience with medial KRs [29]. The last reason for the better survival and lower HR in the subgroup could be the improvement in lateral KR techniques over time, such as proper patient selection, surgical technique, and implant choice [30].

Another reason for the difference between the cohorts could be the use of more mobile bearings in the total cohort than in the subgroup. Mobile bearings dislocate more in the lateral component of the knee. Since 2018 a clear switch from mobile to fixed bearings is seen. Mohammad et al. even concluded that 30% of the revisions in lateral KRs are caused by bearing dislocation, therefore suggesting assessing bearing stability intraoperatively and when unstable implanting a fixed bearing [25]. However, in our study the mobile and fixed bearing ratio is the same in the total cohort and the subgroup. Therefore, this could not be an explanation for the discrepancy in the findings between the subgroup and the total cohort. When the switch to fixed bearings carries through, we expect that the survival rate will increase further as shown in a single-center study by Hariri et al., because revisions for bearing dislocation will occur less frequently [31].

Finally, it could be that medial progression of osteoarthritis occurs in the 2nd decade after the primary procedure. Thus, with the current data we are missing the revisions due to progression of osteoarthritis in the group containing procedures after 2014. We consider this a possible explanation, because in the subgroup only 1.5% had a revision due to progression of osteoarthritis, while in the total cohort 5.3% had a revision because of progression of osteoarthritis. However, of the 48 lateral KRs in the total cohort that were revised because of progression of osteoarthritis, only 6 were revised at 10 years or later. A study by Harrington seems to explain this in terms of loading patterns in the knee [32]. The center of joint pressure is located mostly in the medial compartment of the knee during walking, which could explain progression of osteoarthritis at an earlier stage than in patients with a medial KR.

### Limitations

First, we were not able to include several confounders in our model such as the preoperative indication for TKR (namely varus or valgus deformity). TKRs performed for valgus deformity are generally more difficult; this is actually the group we are interested in for comparison with lateral KR. However, the present comparison of lateral KRs vs the total group of TKRs has definite value, because currently no knowledge is available on the survival of the lateral KR in comparison with the TKR in Dutch hospitals. Nevertheless, we are aware of the missing element and the consequences it could have on the outcome. However, we used the best possible statistics to create weighted cohorts. Second, a limitation of the LROI is the missing individual surgeon volume in the database. Therefore, we could only add the hospital volume as a possible confounder. This is mainly a limitation because most register studies report surgeon volume instead of hospital volume. Furthermore, the implant design will be assessed as a factor that has impact on the outcome of the lateral KR.

### Conclusion

We showed a significantly higher revision rate for lateral KRs compared with TKRs. Caution is needed with the interpretation of the results and a future study comparing lateral KR and TKR for valgus knee osteoarthritis is necessary.

## Notes

IKvO: study design, data analysis, statistics, and drafting of manuscript. LvB: study design, data analysis, and revision of manuscript. AH: interpretation of data, and revision of manuscript. GK: revision of manuscript. PM: data analysis and statistics, interpretation of data, revision of manuscript. LvS: data acquisition, and revision of manuscript. RvG: interpretation of data, and revision of manuscript.

Handling co-editors: Per Henrik Randsborg and Robin Christensen

*Acta* thanks Arild Aamodt, Mona Badawy, and Lars Harald William Engseth for help with peer review of this manuscript.
